# Double-Bladed Scalpel in Mohs Micrographic Surgery

**DOI:** 10.5402/2012/617314

**Published:** 2012-12-19

**Authors:** Ofer Arnon, Vasileios A. Pagkalos, Arsinoi A. Xanthinaki, Eldad Silberstein

**Affiliations:** ^1^Center of R&D in Plastic Surgery, Soroka University Medical Center, Ben-Gurion University of the Negev, P.O. Box 151, 84101 Beer-Sheva, Israel; ^2^Division of Plastic and Reconstructive Surgery, Soroka University Medical Center, Ben-Gurion University of the Negev, P.O. Box 151, 84101 Beer-Sheva, Israel

## Abstract

Mohs micrographic surgery is a tissue-sparing technique that allows for excision of cutaneous tumors under complete microscopic margins control. Mohs surgery boasts high cure rates and maximum tissue conservation. We introduce the double-blade scalpel in Mohs surgery as a timesaving and easy way to harvest tissue strips of uniform width and therefore increase the intraoperative efficiency of the procedure.

## 1. Introduction

 The incidence of both nonmelanoma (NMSCs) and melanoma skin cancers (MSCs) has been increasing over the past decades. NMSCs represent the most common cancer in developed countries, accounting for more than two million cases per year in United States [1].

The treatment options for skin cancers are divided to nonsurgical and surgical. The nonsurgical treatment approach includes radiotherapy, topical therapy (5-fluorouracil-5FU), intralesional administration of interferon, photodynamic therapy, chemotherapy, and oral retinoids [2–6]. The surgical techniques can be destructive (curettage and cautery/electrodesiccation, cryosurgery, and carbon dioxide laser) or excisional (excision with predetermined margins and Mohs micrographic surgery) [2, 4]. The decision of treatment is based upon the type and stage of the skin cancer, the size and topography of the lesion, and the age and overall health condition of the patient. The nonsurgical and the destructive surgical treatments rely on clinical assessment of the tumor's extent and lack pathologic verification of clear surgical margins [4]. Excisional surgical techniques allow for a histologic evaluation of the skin margins and are therefore used for the majority of skin cancers [4, 7].

Although most frequently used, the conventional excision with predetermined margins has a limited capacity of evaluating the surgical margins due to the use of vertical sections (breadloaf and breadloaf-cross methods) in the histological evaluation [4, 8]. The Mohs micrographic surgery (MMS), on the other hand, aims to assess 100% of the peripheral (oblique sections) and deep (horizontal sections) surgical margins [4].

MMS has two main objectives: to remove all of the tumor roots by histologically confirming tumor-free margins and to create the smallest possible defect by sparing tissue uninvolved by the tumor [9]. On average, two stages are required to completely remove the majority of tumors. In the first stage, a peripheral vertical incision is made and a 1-2 mm margin is taken around the tumor for the histological evaluation [4]. The specimen is microscopically examined and any positive tumor margins are marked on a map drawn. The following additional excision of the second stage is based on these markings. 

In order to simplify the excision of accurate peripheral margins during Mohs surgery, we introduce the use of a double-bladed scalpel (DBS) technique.

## 2. Patients, Materials and Methods

### 2.1. Surgical Technique

**Figure 1:**
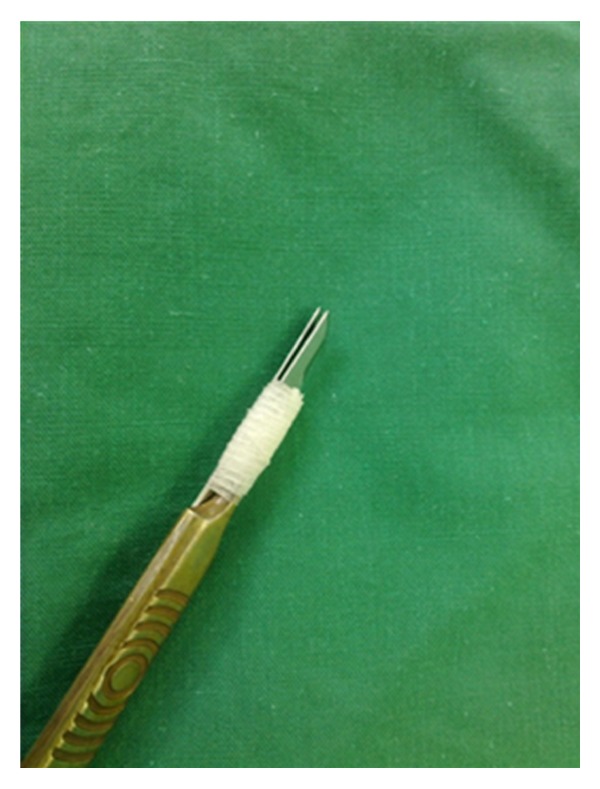
Two separate 15-blade scalpels joined tightly together with a sterile tape on a blade holder resulting in a double-blade scalpel.

**Figure 2:**
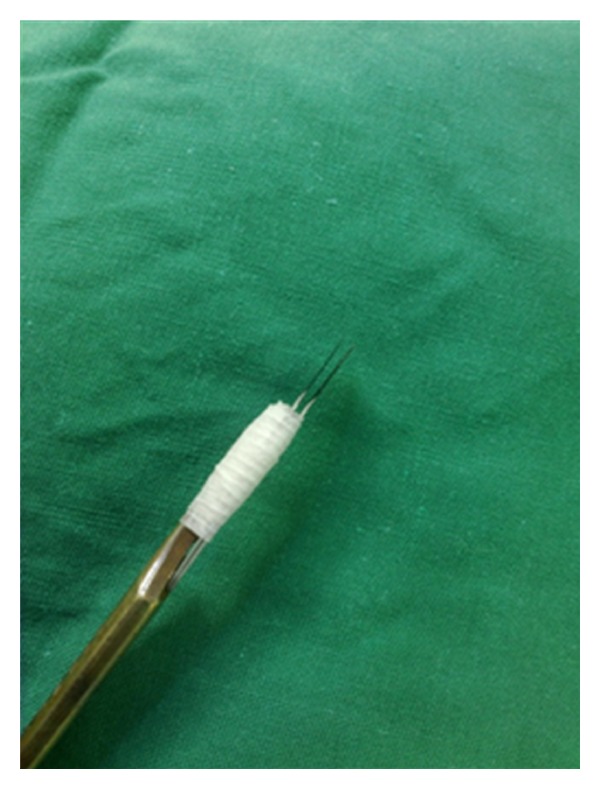
Two separate 15-blade scalpels joined tightly together with a sterile tape on a blade holder resulting in a double-blade scalpel.

**Figure 3:**
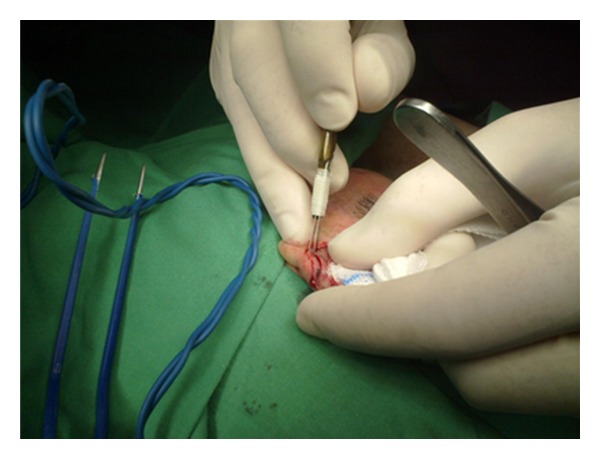
The DBS is used for the 90° vertical incision around the tumor.

**Figure 4:**
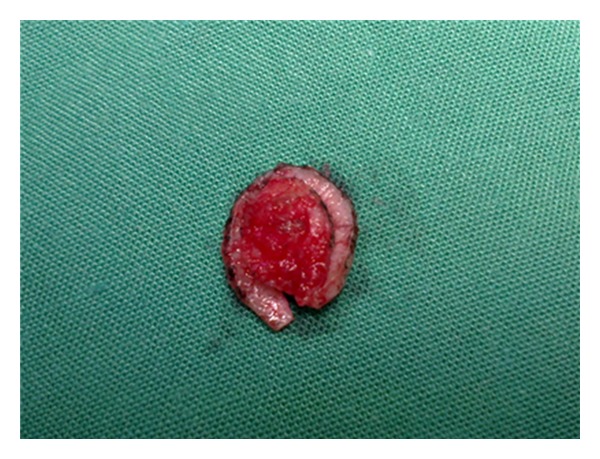
The excised lesion is a central skin piece surrounded by a peripheral skin strip of uniform width.

**Figure 5:**
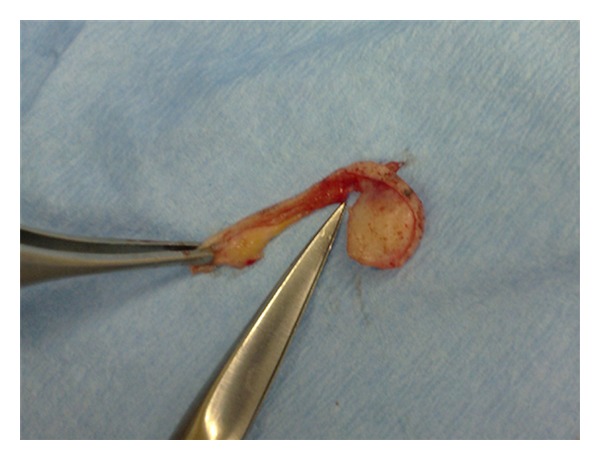
The peripheral skin is cut by seizures.

**Figure 6:**
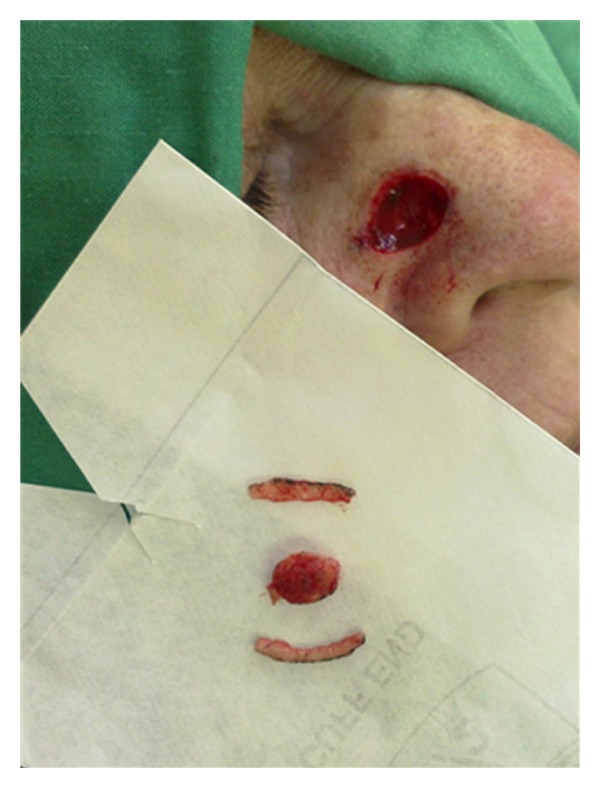
The tumor specimen is divided into pieces: central and peripherals.

Initially, the clinical margins of the tumor are marked with a surgical marking pen and local anesthesia is administered. To assembly the DBS, we use two separate 15-blade scalpels joined tightly together with a sterile tape on a blade holder, allowing for a 1-2 mm interblade gap, as seen in Figures 1 and 2. The interblade gap is easily adjusted according to surgeons preference by changing the number of turns and the position of the sterile tape on the blade holder. The DBS is used for a 90° vertical incision around the tumor (Figure 3) down to a deep dermis level. Then one blade is removed and the excision is completed at the peripheral cut and deep aspect of the specimen, resulting in a central skin piece surrounded by a peripheral skin strip of uniform width (Figures 4 and 5). The tumor specimen is next divided at the side table into one central and two or more peripheral pieces (Figure 6) and accurately mapped and marked with ink for proper orientation. The harvested tissue is then embedded in optimal cutting temperature compound and frozen sections are prepared. Sectioning and staining of the tissue is performed and histological evaluation followed.

### 2.2. Results

Between November 2011 and May 2012, 170 patients with cutaneous tumors underwent Mohs micrographic surgery using the double-blade scalpel technique. The gender distribution consisted of 106 men and 64 women with age raging from 20 to 99 years old (mean 71 years old). 56 patients (32.9%) had positive tumor margins. All positive margins were reexcised with negative results. 46 patients (27%) underwent a two-stage MMS, 7 patients (4.1%) underwent a three-stage MMS, and 3 patients (1.76%) underwent a four-stage MMS. All pathological specimens resulted with accurate uniform easy to examine peripheral margins. 

## 3. Discussion

Surgical excision techniques are the treatment of choice for MSCs and NMSCs. Indications for Mohs surgery include large size of tumor (>2 cm in diameter), incompletely excised tumor, location of the tumor in areas with high local recurrence rate, location of the tumor in areas where tissue conservation and high cure rate are important, tumor with indistinct clinical margins, tumor of aggressive histologic subtype, and tumor arising in irradiated skin or in chronic scars [4]. MMS is a tissue-sparing technique that allows for the excision of tumors under complete microscopic control but, on the other side, requires advanced skills to ensure its efficiency and integrity.

The DBS was first introduced by Coiffmann in 1977 as a harvesting tool for donor strips used in hair transplantation [10]. Schultz and Roenigk used a DBS to cut a strip of tissue around basal cell carcinomas. The strip represented the lateral borders of the excision allowing for a better pathology evaluation [11]. Bowen and Charnock used a DBS to excise surgical scars. They used two parallel blades to cut simultaneously, ensuring that the wound edges will fit together perfectly, despite any deviations resulting from hand movements or difficulties in excising the scar [12]. Cernea et al. presented a study on the reliability of using DBS for the comprehensive excision of surgical margins in large tumors of the head and neck. The preliminary findings of their study showed that the use of DBS is beneficial in harvesting skin and soft tissue margins of large head and neck tumors [13]. The DBS has also been used as an alternative for complete histological margin control in hospitals where it is difficult to perform Mohs surgery. A number of basal and squamous cell carcinomas were excised with a DBS and the tissue strips proceeded to intraoperative histological evaluation. The authors managed to obtain good surgical margin control and no local recurrences were reported [14]. Moossavi et al. were the first to introduce the use of DBS in Mohs surgery. They presented a case of a large dermatofibrosarcoma protuberans (DFSP) where margin control was essential. While Mohs surgery provided the good peripheral margin control needed, the use of DBS reduced the time required to harvest the margins of the massive lesion [15].

In our study the use of DBS in Mohs surgery is extended to varying sizes of head tumors over a period of 18 months and a total of 170 cases treated. DBS is found to be highly effective in harvesting uniform strips of tissue in all cases. DBS's ease of use allows less experienced surgeons to thoroughly excise the peripheral tissue strips needed for the histological evaluation, minimizing the operation error. In the hands of a more experienced surgeon, DBS is timesaving, a value mostly appreciated in hospitals with long lists for Mohs surgery. 

 In a classic Mohs surgery, the blade inserts the skin beveled at a 45° angle when excising the tumor margins. This allows the epidermis, dermis, and deeper tissue to be cut on the cryostat in a straight line and to be examined in one plane [4]. The use of DBS does not allow for this beveling technique to be performed, and this may be considered as a disadvantage over the single blade. However, it has been reported that the tissue harvested with the beveled single blade may not provide a complete epidermal edge for the histological evaluation [4]. A peripheral 90° vertical incision all around the tumor, as the one made with the DBS, can give a more complete epidermal edge. Surgical margins are therefore better to be histologically evaluated with sections from the peripheral strips and separate horizontal sections of the tumor base [4, 16].

 In our study, we used a sterile tape to secure the position of the two separate 15-blade scalpels on a regular blade holder, leaving a 2 mm interblade gap. The assembly is cheap and easy, and, with gaining experience, it does allow for intraoperative adjustment of the interblade gap. 

## 4. Conclusion

 For cutaneous malignancies, Mohs micrographic surgery offers the distinct advantages of complete microscopic margin control and maximum tissue conservation. The use of the double-bladed scalpel in Mohs micrographic surgery is an easy timesaving way to increase intraoperative efficiency.
